# Effects of low‐frequency pulsed electrical stimulation at the common peroneal nerve on chronic refractory wounds of the lower limb: A randomized controlled trial

**DOI:** 10.1002/hsr2.70023

**Published:** 2024-09-08

**Authors:** Yu Ma, Chuan He, Youhui Gong, Lifang Qian, Qian Lu, Jinhua Li, Li‐Juan Zong, Jie Song, Zhifei Yin, Ying Shen

**Affiliations:** ^1^ Rehabilitation Medicine Center The First Affiliated Hospital of Nanjing Medical University Nanjing China; ^2^ Department of Rehabilitation Medicine The Affiliated Jiangsu Shengze Hospital of Nanjing Medical University Suzhou China; ^3^ Oncology Center The Affiliated Jiangsu Shengze Hospital of Nanjing Medical University Suzhou China; ^4^ Department of Rehabilitation Medicine Zhongda Hospital of Southeast University Nanjing China

**Keywords:** chronic refractory wounds, common peroneal nerve, electrical stimulation, wearable device

## Abstract

**Background and aims:**

Electrical stimulation (ES) has been shown to substantially enhance the quality of life by alleviating pain in patients with chronic wounds. This study aimed to observe the effects of low‐frequency pulsed wearable ES at the common peroneal nerve on chronic refractory wounds of the lower limb.

**Methods:**

Forty‐eight participants were randomly divided into control group (*n* = 24) and treatment group (*n* = 24) in this study. The control group received standard wound care (SWC) exclusively, whereas the treatment group was administered both SWC and the wearable low‐frequency ES targeting the common peroneal nerve. Measurements of wound area, pain intensity, wound status, and quality of life scores were systematically recorded both before and after 4 weeks treatment.

**Results:**

After 4 weeks of intervention, the percentage area reduction was significantly higher in the treatment group compared to the control group (*Z* = −3.9, *p* < 0.001), and the healing rate of the treatment group was significantly higher than that of the control group (33% vs. 4%). Moreover, the visual Analog Scale for Pain score (*β* = −0.65, *p* = 0.019), the Bates‐Jensen Wound Assessment Tool score (*p* < 0.05), and the questionnaire on quality of life with chronic wounds (Wound‐Qol) score (*β* = −4.23, *p* = 0.003) were significantly decreased in the patients in the treatment group compared to the control group.

**Conclusion:**

The wearable low‐frequency pulsed ES at the common peroneal nerve for the treatment of chronic refractory wounds showed significant improvement and were far superior compared to SWC. Future research should broaden its scope to include a diverse range of wound types and benefit from collaboration across multiple research centers.

## Key messages

- Traditional wound dressings are currently inadequate for addressing chronic wound closure.
- The wearable electrical stimulation (ES), an economical and noninvasive form of physical therapy, can be beneficial in facilitating the healing process of chronic wounds.
- The purpose of this study was to observe the effects of wearable low‐frequency pulsed ES at the common peroneal nerve on chronic refractory wounds of the lower limb.
- The wearable low‐frequency pulsed ES at the common peroneal nerve for the treatment of chronic refractory wounds showed significant improvement and were far superior compared to standard wound care.

## 1 INTRODUCTION

Chronic wounds are injuries that fail to heal timely and systematically despite receiving standard care for a minimum of 4 weeks, leading to challenges in restoring the skin's structural and functional integrity.^1^ Non‐healing wounds can lead to several complications, such as infection, pain, and a diminished quality of life^2^ thereby creating a significant psychological and financial burden for individuals. Traditional wound dressings, owing to the frequent need for changing the dressing and the non‐conducive nature of blood circulation and granulation tissue growth, among other factors, are currently inadequate for addressing chronic wound closure.^3^ The aforementioned limitations have spurred increasing interest in complementary therapies.^4^ The skin battery theory, proposed by Barker et al. (1983), has garnered more attention towards the potential of electrical stimulation (ES) in promoting wound healing.^5^ According to in vivo studies, ES may expedite wound healing by promoting angiogenesis,^6^ reducing oedema,^7^ and increasing peri‐wound microcirculatory blood flow.^8^, ^9^ Moreover, ES has been shown to substantially enhance the quality of life by alleviating pain in patients with chronic wounds.^10^

Currently, the primary forms of ES utilized in wound management are direct current, alternating current, and pulsed ES.^3^ Among these, unidirectional pulsed current is more commonly used because it mimics endogenous electric fields and avoids skin damage by circumventing thermal effects caused by direct current.^8^, ^11^ According to a meta‐analysis by Khouri and Zheng (2020), pulsed currents were found to be more effective than other currents in promoting chronic wound healing.^12^, ^13^ However, some studies have pointed out that, due to certain design flaws, such as various device parameters, non‐randomized controlled trial, and many other factors in the existing studies, the efficacy of wound healing was not clear.^14^ In addition, the use of conventional pulsed current devices, which typically necessitate placement near the wound, heightens the risk of bacterial invasion and presents inconveniences in instrument use, thereby hindering patient adherence to long‐term treatment. Therefore, an ideal novel ES method should be noninvasive, portable, cost‐effective, and minimally disruptive to the user's daily activities.^15^ Recent studies have shown that home‐based ES was beneficial to the healing of diabetic foot ulcers.^16^

The self‐adhesive neuromuscular wearable device used in our study, featuring low‐frequency unidirectional pulses, noted for its simplicity of operation, portability, non‐invasiveness, and patient tolerability, especially suitable for home‐based treatment. The device consists of a self‐adhesive patch and a rechargeable current‐emitting unit, delivering a current frequency of 1 Hz, a current intensity ranging from 0 to 54 mA, and a pulse width ranging from 25 to 560 µs. The wearable device should be applied to the lateral side of the knee at the head of the fibula. At this location, the common peroneal nerve can be stimulated more easily, causing an ankle dorsiflexion action that produces a muscular pumping effect in the lower extremities and promotes blood flow to the wound bed and surrounding area.^17^ In a separate study, Harris et al. employed the self‐contained stimulation device in treating venous leg ulcers and noted a beneficial effect on wound healing.^18^ However, the majority of studies involving this device are constrained by the absence of a control group, necessitating further controlled clinical trials to confirm its efficacy. Therefore, this randomized controlled trial aimed to compare the differential curative effects of low‐frequency pulsed ES combined with standard wound care (SWC) versus SWC alone.

## 2 MATERIALS AND METHODS

### 2.1 Study design and participants

This study constitutes a prospective, randomized controlled trial aimed at evaluating the clinical efficacy of low‐frequency pulsed ES for chronic lower limb wounds. Forty‐eight patients with chronic wounds were randomly allocated into the control group and the treatment group (24 participants in each group), following the principle of Block randomization.

We included patients with chronic wounds treated in the outpatient wound care department at the Affiliated Jiangsu Shengze Hospital of Nanjing Medical University.

### 2.2 Inclusion and exclusion criteria

Inclusion criteria: (1) Aged 18‐80 years (male or female); (2) Chronic wounds in the lower limbs (wounds that have not recovered after receiving conventional treatment for at least 4 weeks); (3) The ankle brachial index (ABI) range is 0.9–1.3 (to exclude peripheral arterial disease and ensure adequate blood flow around the wound^19^); (4) The wound area ranges from 0.5 to 22 cm²; (5) Informed consent and voluntary participation.

Exclusion criteria: (1) ES devices used in the last 6 months; (2) Pregnancy; (3) Presence of a pacemaker; (4) Multiorgan failure; (5) Malignancy; (6) Systemic diseases; (7) Severe hypertension; (8) Blood disorders; (9) Immunosuppressant/hormone medication use; (10) Severe cognitive/language impairments or incooperation; (11) The skin on the affected side of the fibular head is broken, dermatitis, etc., which is unsuitable for placing the patch; (12) With no foot movement even at the device's maximum setting or beyond the tolerance threshold.

### 2.3 Ethics

The study was approved by the Human Research Ethics Committee of the Affiliated Jiangsu Shengze Hospital of Nanjing Medical University (2022‐019‐01). This trial was registered at the China Clinical Trial Center: Chinese Clinical Trial Registry (ChiCTR2300067503). URL: https://www.chictr.org.cn/showproj.html?proj=178760.

### 2.4 Interventions

#### 2.4.1 Control group: Standard wound care (SWC)

The control group received only SWC without any placebo treatment. A multidisciplinary team consisting of clinicians, nurses, and physical therapists collaboratively tailors the wound care strategy to suit each patient's unique situation. The clinician performs a comprehensive evaluation of the wound status, prescribes appropriate medications, and collects pertinent medical history to identify impediments to wound recovery. The therapist develops a targeted physical therapy program for the affected individuals, while the outpatient nurse administers wound dressing changes, nutritional interventions, and manages contributing factors. Reflecting the consensus among experts in chronic wound treatment, various dressing schemes are developed according to the wound conditions of patients.^1^ The wound care program included thorough debridement, cleansing with physiological saline, application of Biaton silver ion alginate dressings and Biaton foam dressings topically, and protection of the skin around the wound with type II medical gauze, concluding with fixation with elastic bandages twice a week for four consecutive weeks.

#### 2.4.2 **Treatment group: Electric stimulation (ES)** + **SWC**

**Figure 1:**
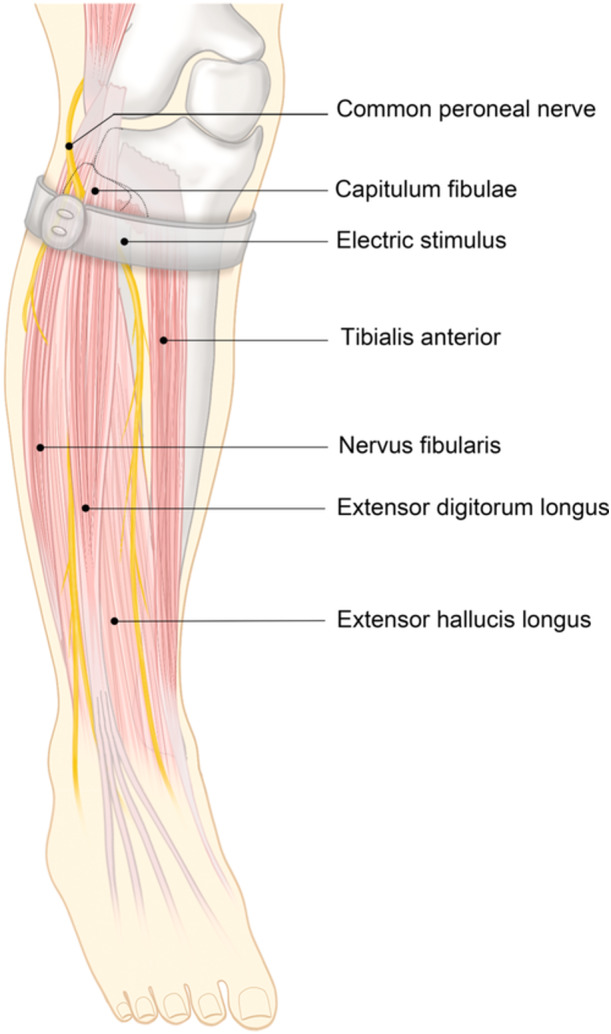
The position of the electrode patch, and the muscles innervated by the common peroneal nerve and its branches.

The treatment group received wearable ES based on the control group. Electrostimulation therapy utilizes a lightweight (45 g), wearable, and rechargeable neuromuscular stimulator (CS‐200P/CS‐200C, Zhejiang Dno Medical Technology Co., Ltd., China). This device emits a low‐frequency unidirectional pulse with a 1 Hz frequency, 54 mA maximum intensity, and ten adjustable pulse widths ranging from 25 to 560 μs. For treatment, the device's patch's alignment marker is placed around the fibular head of the affected knee. The intensity of the ES was set at 54 mA, and the pulse width was adjusted to elicit obvious foot movement. This method involves stimulating the common peroneal nerve on the affected side, leading to the contraction of the innervated muscles and initiating a muscle pump effect in the lower limbs (Figure 1). As the stimulator is placed at the fibular head, distant from any wound site, it eliminates the need to expose wound dressings, thus enabling patients to administer the treatment independently at home. Physical therapists instruct all participants in the device's operation, inform them of potential issues and remedies, and regularly replace the electrostimulation patches. The treatment was administered for 60 min per session, three sessions daily, for four consecutive weeks.

### 2.5 Outcome measures

Relevant medical information was documented after obtaining consent from the participants, and the purpose of this documentation was clearly communicated to the patients. Evaluations of all participants were conducted at baseline, as well as two and 4 weeks post‐intervention, encompassing assessments of wound area, pain, wound status, and quality of life. The wound area was measured using a disposable wound measuring tool (Coloplast, China Medical Supplies, Beijing, China), and photographs of the wounds were captured using a digital camera.

#### 2.5.1 Primary outcome

Percentage Area Reduction (PAR): Recognizing that younger subjects and smaller ulcers generally heal more rapidly, PAR was employed as the primary outcome measure. PAR is a reliable metric for predicting whether a wound will heal within a given timeframe, regardless of the wound's initial size. Higher PAR values indicate a more rapid healing trajectory.^20^, ^21^ The formula to calculate PAR is: PAR = [(A0 ‐ A1)/A0] × 100%, where ‘A0’ denotes the baseline wound area, and ‘A1’ denotes the wound area posttreatment.

#### 2.5.2 Secondary outcome

The secondary outcome measures include:

(1) Healing Rate (HR): These metrics were calculated to determine the complete wound healing rate and deterioration rate of the two patient groups after 4 weeks of treatment. HR is calculated as (Number of Healed Wounds/Total Number of Wounds) × 100%.
(2) Visual Analog Scale (VAS): Pain experienced by patients at rest before each dressing alteration was documented in both cohorts. Participants self‐evaluated their discomfort using the VAS. The VAS consists of a 10 cm line, where “0” indicates no pain and “10” indicates the worst pain ever experienced by the patient.
(3) Bates‐Jensen Wound Assessment Tool (BWAT)^22^: The BWAT was utilized to evaluate the wound status of patients in both groups. The BWAT comprises 15 items, of which two (wound location and shape) do not contribute to the overall score. The 13 scoring items encompass wound size, depth, edges, undermining, types and number of necrotic tissues, exudate type and amount, peri‐wound skin color, peripheral tissue oedema, peripheral tissue induration, granulation tissue, and epithelialization. Each item receives a score ranging from 1 to 5 based on its severity, where 12 items are scored in proportion to their severity, and the ‘epithelialization’ item is scored inversely relative to the extent of epithelialization. The total BWAT score is derived from the sum of the scores of the 13 scoring items. Scores ranging from 1 to 5 indicate tissue health, while scores between 6 and 12 suggest tissue healing. Throughout the course of wound healing, a BWAT score approaching 13 suggests a healing trajectory, whereas a score nearing 60 suggests a deteriorating condition.
(4) Questionnaire on Quality of Life with Chronic Wounds (Wound‐Qol): We used the Wound‐Qol questionnaire to record the quality of life of the two groups of patients before and after treatment.^23^ This questionnaire encompasses four dimensions (physiological, psychological, daily life, and economic) with a total of 17 items. Each item is graded on a scale of 1 to 5, accumulating a maximum of 85 points and a minimum of 17 points. A higher score indicates a poorer quality of life.

### 2.6 Adverse events

ES therapy exhibits minimal adverse effects, with contact dermatitis being the predominant issue, characterized by symptoms such as itchy skin, redness, and skin protrusion. Given that the ES device used in this study is a self‐adhesive, wearable, and home‐based ES device, patients are instructed to use it at home under physician supervision. Consequently, both the intensity of ES and the duration of treatment must adhere to the physician's prescribed guidelines. Adhering to standard operating procedures is expected to prevent additional adverse events. In the event of dermatitis, ES will be immediately discontinued, and a cold compress applied to the affected area. Additionally, local ultraviolet therapy will be administered, contingent on the extent of skin damage, 24 h post‐occurrence.

### 2.7 Randomization

**Figure 2:**
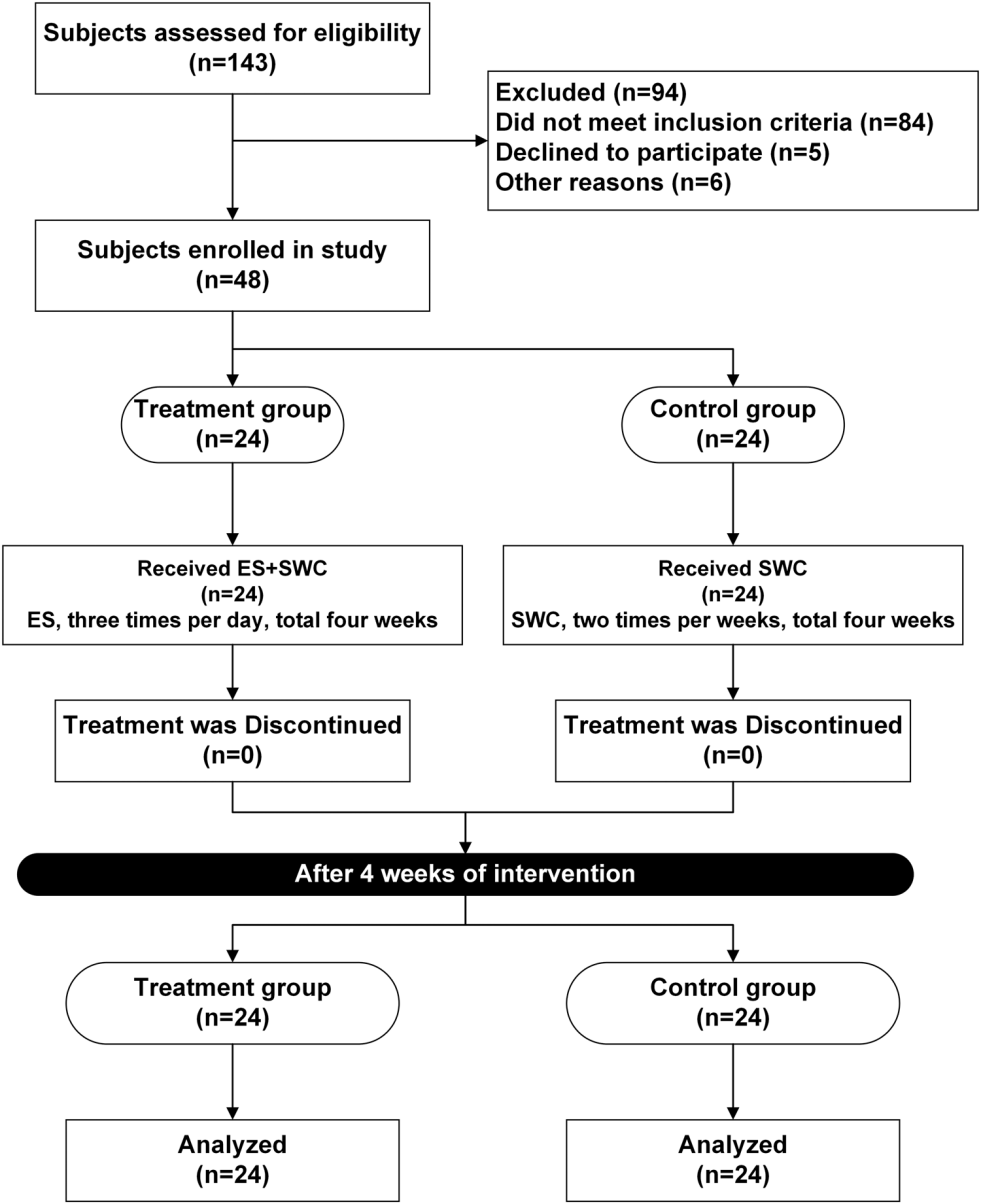
Flowchart of patient enrollment and treatment. ES, electric stimulation; SWC, standard wound care.

Participants were allocated to either the treatment group (X) or the control group (Y) using block randomization. To achieve a final sample size of 48 subjects, four cases were selected for each of 16 designated zones, labeled A through P. Each zone group comprised six permutations of four subjects, namely: XXYY, XYXY, XYYX, YXXY, YXYX, and YYXX. These six permutations were randomized through the generation of a string of random integers (example: 842832470294688141387111418762632) using the website www.random.org. Numbers between 1 and 6 from this string (example: 42324024614131114162632) were selected and organized into blocks corresponding to the random numbers. Subsequently, participants were assigned to the various block groups based on their order of enrollment. This randomization process was conducted by individuals not engaged in the rest of the trial. Participants adhered to the interventions as depicted in Figure 2.

### 2.8 Blinding

Individuals responsible for evaluation and statistical analysis were blinded to the aforementioned group classifications. Participants were informed about either the standard care protocol or the supplementary electric stimulation intervention. After meeting the inclusion criteria and providing informed consent, the assessor conducted an initial assessment and then promptly exited the study. A separate allocator was responsible for communicating the final group allocation. The statistician's role was confined to receiving data from both groups, reflecting their limited participation in the trial.

## 3 STATISTICAL ANALYSIS

First, all data were analyzed for normality and variance using chi‐squared tests. Descriptive statistics were employed to determine the mean and standard deviation for data conforming to a normal distribution, and the median (quartiles) for non‐normally distributed data. Second, we performed between‐group comparisons of PAR, Wound area, VAS, BWAT, and Wound‐Qol at baseline using t‐tests for normally distributed data and non‐parametric tests for non‐normally distributed data. Finally, we compared pre‐ and post‐intervention data between the two groups using repeated‐measures ANOVA for normally distributed data, and generalized estimating equation (GEE) modeling for non‐normally distributed data. All tests were two‐tailed, with *p* < 0.05 considered statistically significant. The Bonferroni method was applied to correct for multiple comparisons to mitigate the increased probability of type I errors.

First, all data were analyzed for normality and variance using chi‐squared tests. Descriptive statistics were employed to determine the mean and standard deviation for data conforming to a normal distribution, and the median (quartiles) for non‐normally distributed data. Second, we performed between‐group comparisons of PAR, Wound area, VAS, BWAT, and Wound‐Qol at baseline using t‐tests for normally distributed data and non‐parametric tests for non‐normally distributed data. Finally, we compared pre‐ and post‐intervention data between the two groups using repeated‐measures ANOVA for normally distributed data, and generalized estimating equation (GEE) modeling for non‐normally distributed data. All tests were two‐tailed, with *p* < 0.05 considered statistically significant. The Bonferroni method was applied to correct for multiple comparisons to mitigate the increased probability of type I errors.

## 4 RESULTS

Forty‐eight patients were ultimately included in the trial, with 24 cases each group. All subjects completed this study. There was no statistical difference in the general information of patients (Table 1).

**Table 1:** Demographic characteristics of participants.

Characteristics	Treatment group (*n *= 24)	Control group (*n* = 24)	*p* Value
Age (years)^a^	57.96 ± 13.28	60.21 ± 12.12	0.543
Sex (female/male)^b^	7/17	9/15	0.540
Weight (kg)^a^	65.87 ± 10.37	63.13 ± 7.21	0.294
ABI^a^	1.09 ± 0.09	1.07 ± 0.08	0.243
Smoking (no/yes)^b^	14/10	13/11	0.771
Wound duration (weeks)^c^	5 (4, 5)	4 (4, 5)	0.549
VAS (pre‐intervention)^c^	6 (5, 6.75)	6 (4, 7)	0.246
Wound‐Qol (pre‐intervention^c^	42 (36.5, 48.75)	42.5 (38.25, 46.75)	0.528
Type of wound^b^			
Traumatic	15/24	14/24	0.768
Surgical	4/24	5/24	0.712
Venous	4/24	3/24	0.683
Diabetic	1/24	1/24	1

^a^ ANOVA.

^b^ Chi‐square test.

^c^ Mann‐Whitney.

### 4.1 PAR and Wound Area

**Figure 3:**
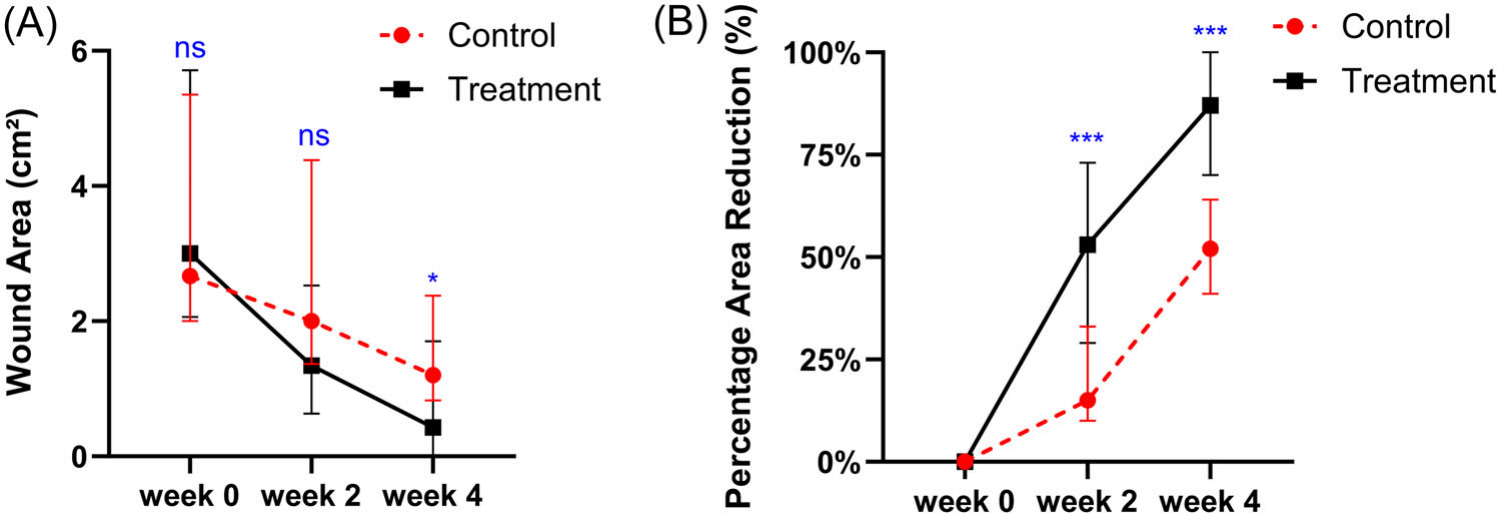
Changes in mean Wound area (A) and Percentage Area Reduction (B) in patients receiving electrical stimulation and standard wound care. ns: *p* > 0.05, *p ≤ 0.05, **p ≤ 0.01, ***p ≤ 0.001.

After 4 weeks treatment, the wound area did not conform to a normal distribution, so a non‐parametric test was used for the between‐group analysis at baseline, which showed no significant difference in the wound area between the two groups (*p* = 0.599) (Table 3). Analysis of wound area before and after the intervention in both groups using GEE revealed a significant interaction term between group and time (*p* < 0.05) (Table 2). Subsequent simple effects analyses showed that after the intervention, both groups had significantly less wound area than before the intervention (*p* < 0.05) (Table 3). In addition, the wound area was lower in the treatment group than in the control group, and the difference was higher after 4 weeks of treatment than in the second week (the mean difference changed from 0.49 to 0.57) (Table 3). For PAR, as normality was not satisfied, non‐parametric tests were used to assess intra‐ and intergroup differences. The results indicated that after the intervention, the PAR reduction escalated in both groups (*p* < 0.001), and the treatment group demonstrated superior PAR at both time points compared to the control group (*p* < 0.001) (Table 4, Figure 3).

**Table 2:** GEE model effects.

Variables	β (95% CI)	Waldχ2	*p*
**Wound area**			
Group effect		2.43	0.119
Time effect		88.01	*p* < 0.001
Group × Time effect		6.42	0.040
**VAS**			
Group effect		5.47	0.019
Time effect		34.43	*p* < 0.001
Group × Time effect		3.39	0.184
Group	−0.65 (−1.19, −0.105)	5.47	0.019
Time			
Baseline			
2 weeks	−0.88 (−1.65, −0.12)	5.10	0.024
4 weeks	−1.20 (−2.70, −1.30)	31.47	*p* < 0.001
**BWAT**			
Group effect		8.34	0.004
Time effect		58.24	*p* < 0.001
Group × Time effect		19.26	*p* < 0.001
**Wound‐Qol**			
Group effect		9.14	0.003
Time effect		35.23	*p* < 0.001
Group × Time effect		4.14	0.126
Group	−4.23 (−6.97, −1.49)	9.14	0.003
Time			
Baseline			
2 weeks	−2.76 (−6.40,0.87)	2.22	0.137
4 weeks	−9.53 (−12.90, −6.17)	30.86	*p* < 0.001

Abbreviations: BWAT, The Bates‐Jensen Wound Assessment Tool; ES, electric stimulation; SWC, standard wound care; VAS, visual analog scale; Wound‐Qol, questionnaire on quality of life with chronic wounds.

**Table 3:** Pairwise comparison.

Summary at different time points								
Median (minimum, maximum)					From baseline to 2 week mean (95% CI)		From baseline to 4 week mean (95% CI)	
Wound area		Baseline^a^	2 weeks	4 weeks	Within‐group change	Between‐group difference in change	Within‐group change	Between‐group difference in change
	Control	2.67 (2.01,5.35)	2.00 (1.37,2.00)	1.20 (0.83,2.38)	0.83 (0.07,1.60)*	−0.49 (−1.80,0.82)	2.06 (1.11,3.01)*	−0.57 (−1.40,0.25)
	Treatment	3.00 (2.06,3.00)	1.34 (0.63,1.34)	0.43 (0.00,1.70)	2.08 (1.13,3.03)*		3.39 (2.14,4.63)*	
**BWAT**								
	Control	32.50 (26.50,35.50)	30.50 (25.00,34.75)	25.50 (23.25,32.75)	1.46 (−2.95,5.86)	−0.83 (−4.31,2.64)	3.83 (−0.41,8.08)	−7.38 (−10.21,−4.54)*
	Treatment	34.00 (31.00,37.50)	29.00 (24.25,34.75)	18.50 (15.25,25.75)	5.08 (0.52,9.64)*		14.00 (9.97,18.03)*	

Abbreviation: BWAT, Bates‐Jensen Wound Assessment Tool.

^a^ Comparison of baseline score between the treatment and control group (wound area: *p* = 0.599 BWAT: *p* = 0.123) *p < 0.05.

**Table 4:** Comparison of the PAR between two groups.

Group	2 weeks	4 weeks	z‐value	*p*‐value
Control	15.31% (15.31%,32.75%)	51.92% (40.8%,63.64%)	−3.914	*p*＜0.001
Treatment	52.64% (28.65%,72.86%)	87.03% (69.7%,100%)	−3.985	*p*＜0.001
z‐value	−4.033	−3.9		
*p*‐value	*p*＜0.001	*p*＜0.001		

Abbreviation: PAR, Percentage Area Reduction.

### 4.2 HR

After 4 weeks of intervention, the HR in the treatment group was significantly higher than in the control group, with a 33% of HR in the treatment group compared to 4% in the control group.

### 4.3 VAS

**Figure 4:**
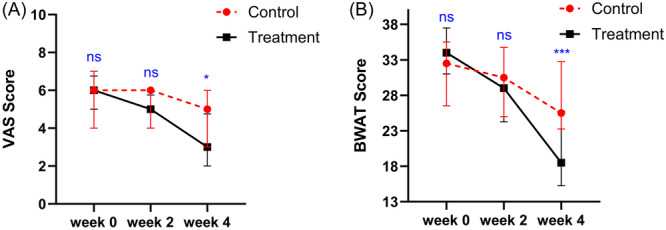
Changes in mean VAS (A) and BWAT (B) in patients receiving electrical stimulation and standard wound care. VAS, visual analogue scale; BWAT, Bates‐Jensen wound assessment tool. ns: *p* > 0.05, *p ≤ 0.05, **p ≤ 0.01, ***p ≤ 0.001.

The VAS did not satisfy the normal distribution, so a non‐parametric test was used for the baseline analysis: There was no significant difference in VAS scores between the two groups at baseline (*p* = 0.246) (Table 1). Subsequently, the results of GEE analysis indicated that the VAS scores were significantly lower at 2 weeks (*β* = ‐0.88, *p* = 0.024) and 4 weeks (*β* = ‐1.20, *p* < 0.001) post‐intervention compared to baseline, and the VAS scores were significantly lower in the treatment group than in the control group (*β* = −0.65, *p* = 0.019) (Table 2, Figure 4).

### 4.4 BWAT

Data analysis revealed that the BWAT did not satisfy the normal distribution, so a non‐parametric test was used to compare the baseline data. Before the intervention, there was no difference in BWAT between the two groups (*p* = 0.123). Modeling using GEE to compare the pre‐ and post‐intervention changes between the two groups revealed a significant interaction term between group and intervention time (*p* < 0.05) (Table 2). Subsequently, a pairwise comparison of the interaction terms was conducted. After both 2 and 4 weeks of treatment, the BWAT of the treatment group were significantly improved compared to the pretreatment period (*p* < 0.05, respectively), and the control group showed no significant improvement. The difference between the groups was only statistically significant after 4 weeks of treatment (*p* < 0.05) (Table 3, Figure 4).

### 4.5 Wound‐Qol

**Figure 5:**
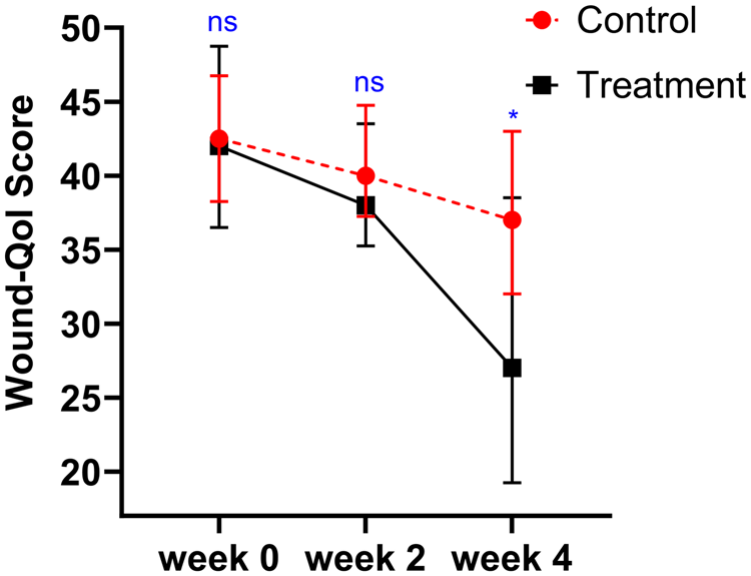
Changes in mean Wound‐Qol scores in patients receiving electrical stimulation and standard wound care. Wound‐Qol, questionnaire on quality of life with chronic wounds. ns: *p* > 0.05, *p ≤ 0.05, **p ≤ 0.01, ***p ≤ 0.001.

The two groups had no difference in Wound‐Qol at baseline (*p* = 0.528). The corresponding GEE model was then constructed, the Wound‐Qol scores of both groups decreased over time and showed a significant difference after 4 weeks intervention (*β* = −9.53, *p* < 0.01), and the treatment group had significantly lower scores than the control group (*β* = −4.23, *p* = 0.003) (Table 2, Figure 5).

### 4.6 The relationship between changes in VAS and Wound‐Qol

**Figure 6:**
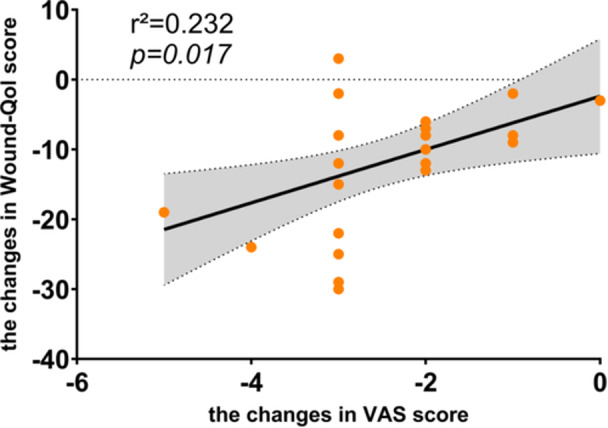
Correlation between VAS and Wound‐Qol score in the treatment group after four weeks of intervention. VAS, visual analogue scale; Wound‐Qol, questionnaire on quality of life with chronic wounds.

After 4 weeks of intervention, the change in VAS was significantly positively correlated with the change in Wound‐Qol in the treatment group r^2^ = 0.231, *p* = 0.017 (Figure 6).

## 5 DISCUSSION

Our research supported the use of wearable home ES devices as a convenient and effective treatment for enhancing chronic wound healing. The findings of this study revealed significant improvements in both primary and secondary markers from their baseline values within each group. In the treatment group, PAR, VAS, BWAT, and Wound‐QoL showed significant improvements compared to the control group, particularly PAR, which demonstrated a statistically significant difference after just 2 weeks of clinical treatment.

We observed no significant difference in the wound area between the two groups post 4 week intervention, potentially attributable to the small size of the included wounds (2.67 (2.01,5.35) vs. 3.00 (2.06,3.00)). However, the paramount outcome in wound‐related research remains healing. Importantly, our study demonstrated a significantly higher healing rate in the treatment group compared to the control group (33% vs. 4%, *p* < 0.05). Central to our findings is that the PAR in the treatment group was significantly greater than in the control group at both 2 week (Z = −4.033, *p* < 0.001) and 4 week intervals (Z = −3.9, *p* < 0.001). Additionally, the PAR at 4 weeks was significantly higher than at 2 weeks (*p* < 0.001), suggesting that the combined application of ES and SWC significantly enhances wound healing. As an intermediate outcome, PAR serves as an insightful predictor of subsequent wound healing, potentially saving considerable research time. Compared to other intermediate outcomes, PAR is particularly advantageous for wound clinics due to its straightforward approach in data collection.^20^, ^21^, ^24^ These results align with Harris et al.'s findings,^18^ which also emphasized the effectiveness of low‐frequency ES in targeting the common peroneal nerve, and recorded significant improvements in wound dimensions and healing trajectories both before and after intervention. Although this nerve stimulation device has shown promising results for intractable lower limb ulcers, further randomized controlled trials are necessary to investigate its efficacy thoroughly.^18^, ^25^, ^26^ Kloth's retrospective analysis also indicated that the combination of ES with SWC might accelerate the wound healing process over using SWC alone.^27^

The mechanisms through which ES promotes wound healing are multifaceted. First, ES can enhance local tissue metabolism. In vivo studies have shown that stimulating calf muscle activity through a muscle pump mechanism increases both arterial and venous circulation to the lower extremities and near the wound,^28^ thereby enhancing oxygenation at the injury site.^29^ Second, ES is known to enhance local blood circulation. Clover et al.^30^ have found that ES resulted in increased microvessel density and a notable increase in local blood perfusion. Third, ES may accelerate wound recovery by reducing the edema around the wound,^7^ which possibly due to its direct effect on vasodilation mediated by neuronal or chemical agents.^31^ Furthermore, compared to standard care treatments, ES stimulates the release of Vascular Endothelial Growth Factor, encouraging angiogenesis.^32^ Preclinical studies have demonstrated that cellular activities, such as collagen and DNA synthesis, ATP concentration, and generation of chemotaxic factors, are enhanced by ES.^33^ Moreover, ES speeds up wound healing by promoting reinnervation and neural differentiation,^34^ aiding in the restoration of sensation and pain relief. An optimal ES device ideally should be noninvasive, portable, cost‐effective, and minimally disruptive to a patient's daily routine.^15^ However, many conventional therapeutic devices require the removal of wound dressings or bandages and need professional medical supervision, significantly extending consultation times. The ES device used in our study placed the electrode pad near the affected fibular head on the affected side, away from the wound, thus reducing the risk of infection from direct contact. Additionally, ES therapy can be applied simultaneously with compression therapy, obviating the need for patients to remove their bandages, thereby minimizing the wound's impact on daily activities. Therefore, it presents a more practical alternative to traditional wired electrical systems. Due to the device's comfort and portability, it may reduce the length of medical visits. Its simplicity also enables at‐home treatment, significantly improving patient compliance and treatment efficacy. This innovative neuromuscular stimulation device emerges as a promising supplemental therapeutic approach, providing substantial benefits to patients with lower extremity ulcers.

In alignment with the aforementioned results, this study also demonstrated that at the conclusion of the 4 week intervention, the BWAT scores of the treatment group were significantly improved compared to those of the control group. The BWAT score, a validated tool, is employed to assess the overall condition of a wound.^22^ Improved wound conditions may be attributed to enhanced blood circulation, increased oxygenation, and edema reduction facilitated by ES. Enhanced circulation near the wound reduces ischemic necrotic manifestations. Vasodilation, which increases blood perfusion, restores skin luminescence. Regulation of exudate results from reduced edema, and increased fibrinolysis, induced by ES, may soften the periphery of the wound.^18^, ^35^

Conversely, although healing outcomes are the primary focus of clinical wound studies, addressing associated pain and its impact on quality of life is crucial.^1^ The findings of our study indicated significant improvements in VAS (*β* = −0.65, *p* < 0.05) and Wound‐Qol (*β* = −4.23, *p* < 0.05) scores in the treatment group compared to the control group. Pain is profoundly correlated with quality of life, frequently acting as a major detractor.^36^ The correlational analysis in our study revealed a significant association between changes in quality of life scores and pain ratings after 4 weeks of treatment (Figure 6). This indicates that enhancements in quality of life may be related to reductions in pain. In contrast to other modalities, the low‐frequency pulsed ES used in our study helps maintain skin pH balance, causes minimal skin damage, and reduces discomfort.^27^ Bosanquet and colleagues observed that low‐frequency ES alleviated vascular pain in patients, even those without wounds.^17^ Simultaneously, Harris et al. determined that the device relieved pain in individuals with venous leg ulcers, encouraging their return to compression therapies.^37^ In our intervention sessions, no subject in the experimental group discontinued compression therapy due to intolerable pain, unlike in the control group. This finding underscores that pain reduction through ES enhances patient adherence, thereby accelerating wound recovery and improving quality of life, creating a self‐reinforcing cycle. The analgesic effects of neuromuscular ES are likely due to gating mechanisms that regulate pain signal transmission through C‐fibers by activating Aβ tactile afferents.^38^ Echoing the significant findings of Clarke Moloney et al.,^28^ our results also suggest that blood flow enhancement is more pronounced when ES is combined with compression therapies, as opposed to compression alone, particularly in patients with venous leg ulcers who experience mobility challenges. This emphasizes the benefits of adhering to combination therapy.

## 6 LIMITATIONS

This study, like other studies, had some limitations. Firstly, the small sample size precluded detailed classification of chronic wounds for specific subgroup analysis, raising uncertainties about the most effective use of the device. In further studies, we will stratify different wound types (such as ulcer, trauma, diabetic foot, postoperative wound, pressure injuries, etc.), to clarify the therapeutic effect under different etiologies better. Secondly, the depth of understanding regarding how the device treats chronic wounds remains limited, particularly its mechanism of action in stimulating angiogenesis and enhancing blood flow and tissue oxygenation. In the follow‐up studies, we will further explore the mechanism by means of pathological examination and blood flow rate assessment. Finally, in our experiment, the initial wound sizes for both groups were relatively small, a factor potentially contributing to the higher healing rate observed in the ES group. Further research should categorize wounds into different groups based on different area and depth, to identify the suitable treatment options. The single ES application mode and waveform used in our trial prevented selection of an optimal modality.

## 7 CONCLUSIONS

Through our randomized controlled trials, the efficacy of the wearable low‐frequency ES device in promoting chronic wound healing has been confirmed. These observed effects are consistent with the general outcomes of ES on the body. Future research should broaden its scope to include a diverse range of wound types and benefit from collaboration across multiple research centers. In addition, the optimal parameters for the treatment of different types of wounds by ES need to be further verified. Whether this type of microcurrent ES can replace traditional ES also needs to be further verified.

## AUTHOR CONTRIBUTIONS

**Yu Ma**: Writing—original draft. **Chuan He**: Writing—original draft. **Youhui Gong**: Data curation. **Lifang Qian**: Data curation. **Qian Lu**: Investigation; methodology; writing—review and editing. **Jinhua Li**: Supervision. **Li‐Juan Zong**: Resources. Jie Song: Project administration. **Ying Shen**: Conceptualization; funding acquisition; methodology; project administration; writing—review and editing. **Zhifei Yin**: Conceptualization; methodology; project administration; writing—review and editing.

## CONFLICT OF INTEREST STATEMENT

The authors declare no conflicts of interest.

## TRANSPARENCY STATEMENT

The lead author Ying Shen, Zhifei Yin affirms that this manuscript is an honest, accurate, and transparent account of the study being reported; that no important aspects of the study have been omitted; and that any discrepancies from the study as planned (and, if relevant, registered) have been explained.

## Data Availability

The data that support the findings of this study are available from the corresponding author uponreasonable request.
